# Attitudes of faculty and residents of surgical specialties towards professionalism at a tertiary care hospital of Islamabad

**DOI:** 10.12669/pjms.35.2.387

**Published:** 2019

**Authors:** Arifa Manzoor, Lubna Ansari Baig, Syed Moyn Aly

**Affiliations:** 1 *Dr. Arifa Manzoor, MBBS, FCPS, MRCS, MHPE. Assistant Professor, Department of General Surgery, Pakistan Institute of Medical Sciences, Islamabad, Pakistan*; 2 *Prof. Lubna Ansari Baig, MBBS, MPH, FCPS, PhD. Pro-Vice Chancellor,*; $$ *Dean APPNA Institute of Public Health, Jinnah Sindh Medical University, Karachi, Pakistan*; 3 *Dr. Syed Moyn Aly, MBBS, MHPE, PhD (Scholar). Department of Medical Education, Jinnah Sindh Medical University, Karachi, Pakistan*

**Keywords:** Professionalism, Attitude, Exploratory Factor Analysis

## Abstract

**Objectives:**

To determine the attitudes of faculty and residents of surgical specialties towards professionalism and to test the validity and reliability of a tool developed in USA for Pakistan.

**Methods:**

An exploratory validation study was carried out at Pakistan Institute of Medical Sciences (PIMS), Islamabad from 01-Aug-2016 to 31-Jan-2017. Penn State College of Medicine Professionalism Questionnaire (PSCOM) was used, being a reliable and valid survey tools. Exploratory Factor Analysis of the inter-correlations of responses for 36 items was done using SPPS v 21 to give a factor solution to reflect the perceptions regarding attitudinal elements.

**Results:**

There were 209 respondents including 172 residents and 37 faculty members. Response rate was 81.32%. Exploratory Factor Analyses of responses gave a seven factor solution of professionalism: accountability, honour and integrity, excellence, duty, altruism, equity and respect. Six of the factors that emerged reflect the six elements of professionalism reported by American Board of Internal Medicine (ABIM), except for ‘equity’, which was the new factor that emerged. Cronbach’s alpha (Internal Consistency Reliability) for each element of professionalism was between 0.88-0.98.

**Conclusion:**

PSCOM is a valid, reliable, feasible and acceptable tool to assess attitudes of faculty and residents towards professionalism in Pakistan. ‘Equity’ emerged as a new factor which needs to be studied further.

## INTRODUCTION

Professionalism is a core competency of physicians. Professionalism is fundamental to medical practice since ancient Greece as evident from the importance given to the professional attitude and behavior in the Hippocratic Oath.^1^,^2^ The Accreditation Council of Graduate Medical Education (ACGME) labeled professionalism as one of its core competencies. Its definition of professionalism includes “a list of attributes and behaviors such as accountability, altruism, commitment to excellence, compassion, integrity, respect, responsiveness, sensitivity to diversity, and sound ethics”.^3^ American Board of Internal Medicine (ABIM)^4^ provided an explicit place for professionalism in the curriculum for post-graduation in internal medicine, and defined the real meaning of professionalism. Their six key elements include altruism, excellence, accountability, duty, honor, integrity and respect for others.^4^

Professionalism impacts patient care, relationships and trust and also public’s perceptions towards doctors. Failing to improve unprofessional attitude despite feedback and remediation is a punishable offence.^1^ Teaching of professionalism is compulsory according to the American Board of Internal Medicine (ABIM), the American Association of Medical Colleges (AAMC), and the Accreditation Council for Graduate Medical Education (ACGME), the European Federation of Internal Medicine and General Medical Council (GMC) of United Kingdom.^2^ Pakistan Medical and Dental Council (PMDC) has proposed to integrate the elements of professionalism in undergraduate as well as post-graduate medical education in Pakistan.^5^,^6^

Professionalism is a multidimensional construct of ethical, social, cultural, relational, and epistemological competencies, thus requiring a variety of tools for its assessment.^7^ Assessment of professionalism is a developing field and needs improvement on the way to generate reliable student, resident and faculty evaluation data. Knowing the perception towards professional attitudes is the first step towards intervention to integrate professionalism in curricula.^1^ American College of Surgeons (ACS) in its code of professional conduct defines a surgeon differently from a competent technician and emphasizes that surgeons’ relationship with society and his patients is a fundamental element of professionalism.^8^ Assessment needs to consider the differences among specialties. Surgical residents have attributes unique to their specialty.^9^

Assessment of professionalism in residents and faculty of surgery and related specialties is needed with exceptional focus to establish the current status in this important discipline. No such study has been reported in our country. To address the gap of understanding professionalism, the aim of our study was to determine the attitudes of the faculty and residents of surgical specialties towards professionalism. This also served to test validity and reliability of a tool developed in USA to assess attitudes towards professionalism in our setup.

## METHODS

This exploratory validation study was carried out at Pakistan Institute of Medical Sciences (PIMS), Islamabad over six months from 1^st^ August 2016 to 31^st^ January 2017 after taking permission from the hospital ethical review committee. All faculty and residents of surgical specialties i.e. General Surgery, Orthopaedics, Neurosurgery, Plastic and Reconstructive Surgery, Paediatric Surgery, Urology, Cardiac Surgery, Maxillofacial Surgery, Otorhinolaryngology, Ophthalmology and Anaesthesiology were briefed about the objectives of the study. Questionnaire was distributed to 53 faculty members and 204 residents of surgical specialties. Faculty and residents not consenting to participate in the study were excluded.

Penn State College of Medicine (PSCOM) Professionalism Questionnaire was developed by Blackall et al. in USA as a reliable and valid tool to measure professionalism.^10^ Penn PSCOM^10^ is a reliable (reliability above 0.7 except for respect which was 0.51) and valid survey instrument to evaluate attitudes reflecting the ABIM’s six elements of professionalism including Accountability, Altruism, Duty, Excellence, Honest and Integrity and Respect in faculty, residents and medical students. A nine member committee was formed at Penn State College of Medicine to design an instrument keeping in view two essential queries: (a) What is professionalism at institutional level? And (b) How does socialization effect its development? Faculty of the committee belonged to both the basic and clinical sciences and was endowed with a basic knowledge of professionalism and was motivated to work on the project. They employed the approach of domain sampling for effective item development and collected 60 items representing the general, frequent and developing interpretations of professionalism found in the literature according to the six listed ABIM elements.^4^ These items were in agreement with an internal document demarcating the goals of undergraduate medical education at PSCOM. Modified Delphi Technique was used to reach a consensus on the most suitable items reflecting each one of the six elements. Redundancy occurring among the items was removed and items whose context matched with the six elements of ABIM professionalism were retained. Each item was reviewed for conceptual fit. Items were deleted when they did not fit appropriately with the respective scale as well as when they could not increase the overall reliability of each scale. 41 out of 60 items were retained after three rounds of deliberation. Each item statement was jointly classified and coded into one of the elements by the task force. It resulted in some elements having more items than the other. Again consensus was developed to retain six items for every element.

PSCOM was distributed after obtaining prior authorization for its utilization from the author. We have used the form for the Clinical Science Faculty (Appendix A) and Residents (Appendix B). First part of the proforma included demographic details like age, gender, etc and modified according to our setup. There were a total of 36 items with six items representing six ABIM’s elements of professionalism i.e. Accountability, Altruism, Duty, Excellence, Honesty and Integrity and Respect for others. All of 36 items were divided into six with every group containing six random items from each of the six elements. The faculty and residents were asked to match the items with their attitudes towards professionalism on a 5 point Likert scale. The 5 point Likert scale included: Never, Little, Some, Much, Great Deal. Maximum score given to every item was 5.

Data was stored and analysed on IBM SPSS version 21.0 for Windows software in terms of various descriptive statistics like frequencies and percentages for qualitative variables like response rate, and gender. Mean and ± Standard Deviation (SD) were used for quantitative data i.e. age, years since training for faculty and years as faculty. We carried out Factor Analysis of the inter-correlations of the responses and Cronbach’s alpha (Internal Consistency Reliability) was measured for all the derived factors.

## RESULTS

We got a total of 209 (172 residents and 37 faculty members filled and returned) questionnaires back out of the 257 distributed giving a response rate of 69.81% for faculty, 84.31% for residents and a combined 81.32% response rate for both. Out of a total of 160 male respondents, 130 were residents and 30 faculty. Among females 42 were residents and 7 faculty members. Faculty who responded were between 37 to 58 years with a mean age of 50.08 ± 6.88. They did their post-graduation from 7 up to 28 years ago with mean time of 17.46 ± 6.08 years, and were serving as faculty in academic positions for the last 5 to 27 years with a mean time of 15.59 ± 6.35 years as faculty. Residents who responded were between 25 to 37 years of age with the mean age of 30.02 ± 2.26 years.

**Fig.1:**
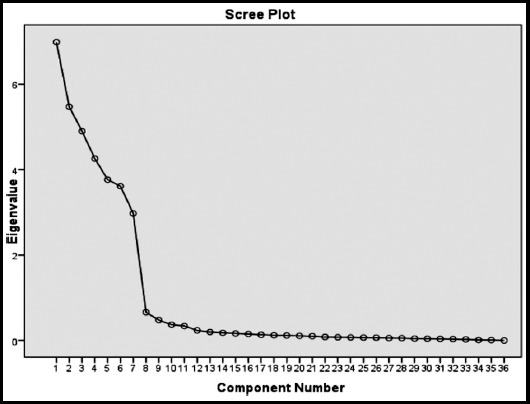
Scree Plot.

We did Principal Component Factor Analysis of the inter-correlations of responses for the 36 items that reflected the ABIM’s six elements of professionalism. Using the Kaiser’s Criterion a meaningful seven factor solution was ascertained with Eigen values greater than 1.0. Final factor structure was confirmed by looking at scree plot given in Fig.1.

We kept Eigen values greater than 1 and got a seven factor solution explaining 88.78% of the variance in the data set. Table-I outlines the total variance. ‘Accountability’ is responsible for 19.39% of the variance, ‘honour and integrity’ for 15.19%, ‘excellence’ for 13.60% , ‘duty’ for 11.82%, ‘altruism’ for 10.45%, ‘equity’ for 10.03% and ‘respect’ contributed the least i.e. 8.26% of the variance as outlined in Table-I.

**Table-I:** Factor Analysis Solution with Total Variance Explained.

Factor	Extraction Sums of Squared Loadings			Rotated Sums of Squared Loadings		

	Total	% of Variance	Cumulative %	Total	% of Variance	Cumulative %
1. Accountability	6.98	19.39	19.39	5.65	15.71	15.71
2. Honora and Integrity	5.47	15.19	34.59	5.16	14.33	30.04
3. Excellence	4.89	13.60	48.20	4.79	13.31	43.35
4. Duty	4.25	11.82	60.03	4.74	13.18	56.54
5. Altruism	3.76	10.45	70.48	4.48	12.46	69.00
6. Equity	3.61	10.03	80.52	3.59	9.98	78.98
7. Respect	2.97	8.26	88.78	3.52	9.79	88.78

***Extraction Method:*** Principal Component Analysis

The extracted factors were compared with the a priori factors i.e. ABIM’s six elements: Accountability, Altruism, Duty, Excellence, Honesty and Integrity and Respect. Table-II outlines the seven factor solution that emerged along with number of item loading for every derived element and estimates of the internal consistency reliability (Cronbach’s alpha). Six of the factors that emerged reflect the six elements of ABIM, except for equity which emerged as new factor. Initially all the six ABIM elements had six items/variables. After conducting Factor Analysis we were able to give a seven factors solution but with a different factor structure. The original elements in our study did not exactly match the ABIM factor solution. However, 23 of the items still matched their original elements while 13 were not able to mirror the ABIM factor solution. Each factor has between 4-6 items which is acceptable as every factor should have at least three items. The Penn State University study also gave a seven factor solution but their factor structure was very variable from the ABIM’s elements and our study. The reliability ranged from 0.88-0.98 which is very high.

**Table-II:** Professionalism Instrument 7 Factor Structure.

Factor 1- Accountability	Items	Cronbach’s Alpha
Works collaboratively and respectfully within a team to the benefit of improved patient care or to the contribution of research	6	0.98
Upholds scientific standards and bases decisions on scientific evidence and experience		
Participates in corrective action processes toward those who fail to meet professional standards of conduct		
Recognizes one’s own limitations		
Demonstrates adaptability in responding to changing needs and priorities		
Discloses conflicts of interest in the course of professional duties and activities		
***Factor 2- Honour and Integrity***		
Represents information and actions in a truthful way	6	0.96
Refusal to violate one’s personal and professional code of conduct		
Reports medical or research errors		
Reports data consistently, accurately and honestly		
Meets commitments and obligations in a conscientious manner		
Acts in ways that show a commitment to confidentiality		
***Factor 3- Excellence***		
Seeks self-improvement	5	0.98
Participates in activities aimed at attaining excellence inpatient care		
Assumes leadership in patient management		
Responds to constructive criticism by working to improve one’s capability in the area criticized		
Meaningfully contributes to the teaching mission of the department and the College of Medicine		
***Factor 4- Duty***		
Assumes personal responsibility for decisions regarding patient care	5	0.98
Takes time to review other colleagues work and provides meaningful and constructive comments to improve it		
Shows a willingness to initiate and offer assistance toward a colleague’s professional and personal development		
Attends faculty meetings, seminars, and student research presentations as a reflection of support		
Promotes the welfare and development of junior faculty		
***Factor 5-Altruism***		
Advocates a patient’s or research subject’s interest over one’s own interest	5	0.97
Shows compassion		
Volunteers one’s skills and expertise for the welfare of the community		
Demonstrates empathy		
Maintains patient/physician relationships that do not exploit personal financial gain, privacy, or sexual advantages		
***Factor 6- Equity***		
Adopts uniform and equitable standards for patient care	4	0.96
Respects the rights, individuality, and diversity of thought of colleagues and students		
Commits to implement cost-effective patient care		
Promotes justice in the health-care delivery system by demonstrating efforts to eliminate discrimination in health care		
***Factor 7- Respect***		
Does not seek to advance one’s career at the expense of another’s career	5	0.88
Respects patient autonomy and helps them make informed decisions		
Is professionally attired in a manner that is respectful of others		
Appreciates and respects the diverse nature of research subjects and/or patients, and honours these differences in one’s work with them		
Avoids offensive speech that offers unkind comments and unfair criticisms to others		

## DISCUSSION

ABIM^4^ a priori categories of accountability, altruism, duty, excellence, honesty and integrity and respect were largely retained by the attitudinal elements that we got after our research except for ‘equity’ which emerged as the new element. Despite sharing the six original elements with ABIM the factor structure of our study was not able to mirror the a priori categories. This difference is likely due to the difference in views held by the medical professionals in our country. This enhances the accuracy of the measurements by our study in our context. The study carried out at Penn State College of Medicine yielded seven elements i.e. accountability, enrichment, altruism, equity, duty, honor and integrity and respect but extracted factors and their structure was different from those of ours as well as from the a priori categories of ABIM.^10^ Blackall et al.^10^ concluded that the ABIM elements may need fine tuning according to their results as they had two new factors called ‘enrichment and equity’. We also suggest that adjustment is needed in the items of the questionnaire to reflect the ideas of respondents in our setup.

‘Equity’ has emerged as the new factor; it is thus thought to represent an important place in professionalism. ‘Equity’ is among the four principles given by World Health Organization to guarantee rights of people towards healthcare.^11^ Principle of equity is one of the humanistic principles, its aim is to make sure that everyone can avail health care services without facing any discrimination.^12^ Equity should be fostered at every level so that every individual has the chance to enjoy optimal health and this chance should never be based on identity, ability or social status.^13^ Number of derived factors does not matter as long as the items cover the most essential features.

ABIM framework was originally developed in USA and is considered to be one of the most reliable frameworks reported to assess attitudes towards professionalism. It was subsequently used by Blackall et al.^10^, Quaintance et al.^14^, and Symons et al.^15^ in their studies at USA. The ABIM framework has also been found useful in other contexts and outside USA by Tsai et al.^16^(Taiwan), Aramesh et al.^17^ (Iran), Suzuki^18^ (Japan) and Al-Eraky & Chandratilake^19^ (Middle East), with minor adjustments. According to Hilton & Slotnick^20^ elements of professionalism are ethical practice, reflection, respect for patients, responsibility for actions, social responsibility, self-awareness and teamwork. Chard et al.^21^ approved appraisal, careers, education, leadership and research teams as tenets of professionalism. Steinert et al.^22^ listed altruism, autonomy, commitment, competence, ethics, honesty, integrity, morality, responsibility to society, responsibility to the profession, self-regulation and teamwork as factors required for professionalism. According to Rogers & Ballantyne (2010)^23^, care for colleagues, collaboration, honesty, probity, reflection, capacity, respect for patients, relationship with patients, responsibility, self-awareness and teamwork are the elements of professionalism. Cronbach’s alpha value ranged between 0.88 to 0.98 for the derived elements of our study. Thus the reported internal consistency of the elements is very high. When we compare our results with those of Blackall et al.^10^ we see that reliability of all factors is between 0.71-0.78 except for ‘Respect’ which is 0.51. This difference may be attributed to the presence of medical students as well as basic science faculty in their data.

The need to assess and teach professionalism is important in surgery like any other specialty.^24^ It has been recommended by College of Physicians and Surgeons of Pakistan to include professionalism in the curriculum of postgraduate education in Pakistan.^25^ Professionalism training should also be provided to all members of the teaching faculty.^8^ One of the strengths of our study was the diverse composition of our resident as well as faculty sample and we propose that our tool can be used for needs assessment before training on professionalism.

### Limitations of the study

Generalizability of findings is restricted as data was collected at one institution only. Repetition of study at multiple teaching institutes can result in the measurement of attitudes towards professionalism on a broader perspective.

## CONCLUSIONS

Academic medicine has enormous responsibility and accountability to strengthen the resolve of doctors to exhibit the utmost level of professionalism in their clinical practice. We can use PSCOM for conducting needs assessment for curriculum development. It can measure attitude towards professionalism and track changes in attitudes over time or after curricular changes. This can also be used to assess medical educators and/or faculty. Cultural context like coming on time etc. as part of professionalism can be added in the items of the questionnaire to enhance its utility in our local scenario. This instrument can also be used for the undergraduate medical students and basic science faculty as needs assessment for curriculum development.
